# Current preventive measures for health-care associated surgical site infections: a review

**DOI:** 10.1186/s13037-014-0042-5

**Published:** 2014-10-11

**Authors:** David M Tsai, Edward J Caterson

**Affiliations:** Division of Plastic Surgery, Brigham and Women's Hospital, Harvard Medical School, 75 Francis Street, Boston, 02115 MA USA

**Keywords:** Nosocomial, Surgical site infections, Health-care associated infections, Hospital-acquired, Preventive measures

## Abstract

Healthcare-associated infections (HAIs) continue to be a tremendous issue today. It is estimated 1.7 million HAIs occur per year, and cost the healthcare system up to $45 billion annually. Surgical site infections (SSIs) alone account for 290,000 of total HAIs and approximately 8,000 deaths. In today's rapidly changing world of medicine, it is ever important to remain cognizant of this matter and its impact both globally and on the individual lives of our patients. This review aims to impress upon the reader the unremitting significance of HAIs in the daily practice of medicine. Further, we discuss the etiology of HAIs and review successful preventive measures that have been demonstrated in the literature. In particular, we highlight preoperative, intraoperative, and postoperative interventions to combat SSIs. Finally, we contend that current systems in place are often insufficient, and emphasize the benefits of institution-wide adoption of multiple preventive interventions. We hope this concise update and review can inspire additional dialogue for the continuing progress towards improving patient care and patient lives.

## Introduction

Since the early beginnings of modern medicine, nosocomial infections or healthcare-associated infections (HAIs) have come hand in hand with any progress in medicine and surgery. Without question, we have come a long way since the days the "good old surgical stink" was lauded. This now gone era was a time when surgeons took pride in their accumulated filth as a mark of their experience and professional status, and would thus regularly operate with bloodstained, unwashed garments [1]. Much of the progress since then is owed to Joseph Lister, an English surgeon who is considered the father of antiseptic surgery. He championed carbolic acid sterilization, hand washing, clean garments and gloves [2]. Later on, the discovery of penicillin in 1928 and its mass production in the 1940s increasingly tipped the scales in our favor. And for some time, it seemed like we were on the brink of victory in the war against HAIs. Yet any sort of celebration was short-lived, for as our antibiotics became stronger and more pervasive, certain strains of bacteria brooded in defiance and soon emerged resistant to our drugs.

Further, as the field of medicine advanced, its very landscape changed—hospitals grew larger, patient lives extended well beyond what was ever thought possible, and the kinds of diseases doctors treated shifted towards that of a chronic nature. This came with consequences that became apparent too late. The unbridled use of antibiotics increased the life expectancy of patients with chronic illnesses, but at the cost of harboring resistant microorganisms. Subsequently, these bugs slowly spread beyond the doors of the hospital until these fugitive strains became part of the normal flora in the community.

This is the matter at hand today, and indeed the implications are enormous, astronomical even, if we fail to remain vigilant. The Centers for Disease Control (CDC) estimated in 2002 that 1.7 million HAIs occur annually and about 1 in 20 hospitalized patients will develop an HAI, of which, 99,000 will result in deaths [3]. In terms of healthcare expenditure, the annual direct cost of HAIs is approximately $28-45 billion [4]. Greater still are the costs to a patient when a seemingly "run-of-the-mill" medical or surgical procedure unexpectedly turns into a fight for his or her life. Such was the case for 34 patients in Harborview Medical Center in 1980 when a man with 35% total-body-surface-area burn was transferred from a burn unit endemic with methicillin-resistant *S. aureus.* Even with standard wound precautions, this antibiotic-resistant *S. aureus* was transmitted to 34 other patients. Ultimately, 27 were infected and 17 of the 34 died [5].

At present we live in a world of unparalleled capability in science, technology, and medicine. Things that were once only imagined in fiction and sci-fi movies are quickly becoming our reality. Not only do we routinely perform heart and lung transplants, but we have entered the realm of face and hand transplantation. Despite all these advances, health-care associated infections have repeatedly proven to remain a formidable force that looms in the background—one that if we don't actively and continually combat can threaten to undo any good we strive to accomplish.

### Definition

Healthcare-associated infection is officially defined by the CDC/National Healthcare Safety Network (NHSN) Surveillance as "a localized or systemic condition resulting from an adverse reaction to the presence of an infectious agent(s) or its toxin(s) [6],[7]. There must be no evidence that the infection was present or incubating at the time of admission to the acute care setting." The "big four," which are the 4 most common types, are urinary tract infections (UTI), surgical site infections (SSI), bloodstream infections (BSI), and pneumonia (PNEU) [6],[7]. SSIs account for roughly 1/3 of all HAIs, and catheter-associated BSIs, catheter-associated UTIs, and ventilator-associated pneumonias account for the remaining 2/3 [6],[7].

SSIs are classified into incisional and organ/space, with specific criteria for each [6],[7]. Incisional is sub-classified into superficial incisional, involving only the skin and subcutaneous tissue, and deep incisional, involving fascia and muscle. Organ/space SSI involves any part of the body that was opened or manipulated during the operation, excluding the incision, fascia, and muscle layers. For a more detailed description of each, including signs/symptoms, please refer to CDC/NHSN criteria [6],[7].

### Goals

The goal of this review is to impress that this issue should be paramount to the daily practice of medicine. To that aim, we provide an update and succinct summary of the literature regarding the etiology of HAIs and highlight some preventive measures that can be successfully implemented, specifically concerning SSIs. We also briefly introduce a novel treatment methodology that our lab has been developing as a potential avenue to combat nosocomial SSIs. Lastly, we want to emphasize the implications of this issue to the healthcare system and to the individual patient.

### Pathophysiology/etiology

The etiology of HAIs is undeniably multifactorial. However, the source of contamination can often be attributed to the endogenous skin flora of either the patient or hospital staff [8]-[10]. There are two categories of flora: resident and transient [9],[11],[12]. Resident flora are microorganisms that normally colonize an individual and live in harmony with the host, usually providing some benefit or protection [9],[11],[12]. Conversely, transient flora are microorganisms picked up from the environment. They often do not survive very long on the host, but are easily transmissible from one to another or back to the environment [9],[11],[12].

Often referred to as the human "microbiota", the resident flora vastly outnumber human host cells by 10–100 times and form a commensal community. Over the past decade, new sequencing technologies and emerging fields such as metagenomics have enabled researchers to begin characterizing this intricate, dynamic micro-ecosystem and its implication on host health and disease [12]-[16]. It is thought that a disruption or alteration of the complex interactions among human cells and endogenous microorganisms can lead to disease states, such as autoimmune, metabolic, infectious, inflammatory, and even psychological disorders. In the realm of HAIs, studies have suggested that the microbiota can act as a physical barrier to colonization and/or keep the potential virulence of some endogenous microorganisms in check [12]-[16]. An insult to host homeostasis whether as a consequence of surgery or another process may in fact disturb the balance of this delicate ecosystem, permitting the overgrowth of specific strains of resident flora with pathogenic potential and/or colonization of transient flora, transmitted by the environment or by hospital staff [12]-[16].

Some of the most common microorganisms that account for HAIs include coagulase-negative staphylococci, Staphylococcus aureus, Enteroccocus species, Candida, Escherichia coli, Pseudomonas, and Klebsiella [17]. There is a significant percentage of HAIs associated with multidrug-resistant pathogens (~16%) [17]. The most common includes Methicillin-resistant Staphylococcus aureus (MRSA), accounting for 8%, Vancomycin-resistant Enterococci faecium (VRE), and Carbapenem-resistant P. aeruginosa [17]. About 25-30% of the community is now colonized by S. aureus and up to 5% are colonized with MRSA [18].

These microorganisms spread through many routes. The most common of which includes contact, air, water, and vehicle.

**• Contact transmission** is by direct contact, and perhaps the most common and easiest way for resident and transient flora to spread to a susceptible patient. It is also the route most easily traced back to healthcare workers and staff. This is often due to improper hand hygiene, poor antiseptic technique, contaminated needles, instruments, or dressings.

**• Air transmission** is often invisible and insidious since microorganisms carried in the form of airborne droplets can travel long distances, especially if ventilation is poor. Coughing and sneezing are common ways these pathogens can become airborne. A proper filtration system in place is helpful to prevent hospital-wide transmission.

**• Water transmission** is an underappreciated route for the spread of pathogens. Contaminated hospital water can cause devastating nosocomial outbreaks. It is estimated that waterborne pseudomonas infections kill 1400 annually in the US [19]. Moreover, opportunistic fungi are also a significant threat, especially to immunocompromised patients [20],[21].

**• Vehicle transmission** is by contaminated surfaces and objects such as food, medications, devices, and equipment. This mode, like contact transmission, is often the cause of cross-transmission among susceptible patients, and can easily cause an outbreak. Objects likely to harbor viable pathogens are known as fomites and includes common day use objects like stethoscopes, marking pens, ties, and ID lanyards. Studies have shown that stethoscopes are commonly colonized with S. aureus and MRSA, and that physicians have poor stethoscope cleaning practices [22]-[24]. Contaminated environmental surfaces are also an important aspect of this mode of transmission. Pathogens easily cross-transmit via bed rails, call buttons, trays, chairs, door handles, tabletops, and also through improperly cleaned equipment such as ultrasound machines or defibrillators.

### Preventive measures

Below, we have compiled a concise summary of the most common preventive measures in the literature. Perioperative measures are broken down into preoperative, intraoperative, and postoperative to highlight the many considerations before, during, and after a procedure. This is not meant to be conclusive, but rather, a quick resource from which a dialogue can be sparked regarding what more can be done to prevent nosocomial SSIs. For a more comprehensive look into grades of recommendations and levels of evidence, please refer to Bosco et al., Savage et al., or Fletcher et al. [25]-[27].

### Hand hygiene

Improved hand hygiene is the most important preventive measure we can take. Yet compliance is often low. Healthcare workers often forget or don't spend enough time washing. The CDC recommends washing for at least 20 seconds or the duration of the "Happy Birthday" song sung twice [28]. The World Health Organization (WHO) has developed a multimodal approach to improve hand hygiene compliance. In a study done during a two-year period in Costa Rica, Italy, Mali, Pakistan, and Saudi Arabia, they implemented their approach and found that overall compliance increased from 51.0% to 67.2% [29]. Their strategy consists of five main components: access, training and education, monitoring and feedback, visual reminders, and creation of culture. The WHO details 5 key moments hand hygiene should be practiced [30]:

1. Before touching a patient
2. Before clean and aseptic procedures
3. After contact with bodily fluids
4. After touching a patient
5. After touching a patient's surroundings

The question of which method of hand-washing is better, traditional hand-scrubbing or hand-rubbing with aqueous alcoholic solution has been studied. The general consensus is that if your hands are visibly dirty, traditional hand-scrubbing with soap and water is best. Otherwise, hand-rubbing with aqueous alcohol is comparable [27],[31]-[34]. A randomized equivalence trial compared the two and looked at a total of 4387 consecutive patients who underwent clean and clean-contaminated surgery. SSI rates were 55/2252 (2.44%) in hand-rubbing versus 53/2135 (2.48%) in hand-scrubbing [32]. However, compliance was much better in hand-rubbing, presumably due to ease and access, compared to hand-scrubbing (44% vs 28%). There was also better tolerance, less skin dryness, and irritation [32].

### Directed antibiotic therapy

With the increased emergence and threat of multi-drug resistant microorganisms, narrow-spectrum, directed antibiotic therapy is imperative. Broad-spectrum antibiotics should be exercised with restraint and only used as an initial temporizing measure until a specific diagnosis of the inciting pathogen is reached. Subsequently, they should immediately be discontinued and substituted with narrow-spectrum antibiotics. Reduction of broad-spectrum antibiotic use can undoubtedly be curtailed with the aid of faster, more reliable molecular diagnostic techniques. Next generation methodologies such as PCR sequencing, sonication, and FISH have shown much promise, and have been proven superior to traditional culturing methods in terms of turnover, sensitivity, and accuracy [35]-[40]. The continued advancement of these molecular tools will revolutionize the way we detect and identify microorganisms, and ultimately permit rapid, tailored antimicrobial therapy from the very get-go.

### Preoperative protocols

There are several preoperative protocols that show promise in decreasing incidence of HAIs. Some protocols, however, have little to no benefit, and energy and resources should therefore be shifted to those that yield a significant difference.

**• Screening for Methicillin-sensitive Staphylococcus aureus and MRSA:**

As mentioned, 25-40% of community is colonized with S. aureus [41]-[43]. The bacteria usually reside in the anterior nares. Carriers have been shown to be at higher risk of staphylococcus infections and are 2-9× as likely to have SSI [41]-[43]. They are also at higher risk of nosocomial blood stream infections and lower respiratory infections [44]-[46].

In 2002, Perl et al. [47] looked to determine if intranasal mupirocin reduced rate of S. aureus infections at surgical sites and prevents other nosocomial infections versus placebo. In this randomized, double-blind, placebo-controlled trial, they randomly assigned patients, both carriers and non-carriers, to either the treatment arm or placebo arm. Their results indicated that the intervention did not significantly decrease rates of S.aureus SSI overall, but did significantly reduce the rate of nosocomial S. aureus among carriers (4.0% vs 7.7%) [47]. This suggested the intervention would be more beneficial for nasal carriers rather than an institutional-wide prophylactic treatment for all patients.

As such, a number of institutions implemented a screening process followed by decolonization. In one randomized, double-blind, placebo-controlled multicenter study, rapid identification of carriers by polymerase chain reaction followed by treatment with mupirocin nasal ointment and chlorhexidine soap reduced nosocomial S. aureus infections. The rate of S. aureus infection was 3.4% (17 of 504 patients) in the treatment group versus 7.7% (32 of 413 patients) in the placebo group [48]. Similarly, a prospective cohort study of total joint replacement patients demonstrated a decrease in SSI rate in intervention patients from 2.7% (20/741) to 1.2% (17/1440) [49].

One of the concerns with prophylactic mupirocin administration is the development of resistance. In their study, Perl et al. [47] only identified 4 isolates resistant to mupirocin. Three were obtained from those not treated with mupirocin. They concluded that a single, short course did not appear to select for resistant isolates.

**• Decolonizing hospital personnel:**

Following the same principle as preoperative decolonization of patients, one preliminary study looked at the effects of decolonizing hospital personnel, specifically the surgical team [50]. Carriers were identified among team members (surgeons, anesthesiologists, nurses) and subsequently treated with intranasal mupirocin. Retrospectively, 1000 consecutive patients had yielded 6% SSI rate. Post-intervention, of 300 consecutive patients, there was a 0% SSI rate [50]. Undoubtedly, more studies are needed, but this preliminary finding is important since healthcare personnel are often at fault in the transmission of microorganisms. Institution-wide screening and decolonization of personnel may be a feasible and successful preventive measure. At present most hospital systems have no screening methodology for employees with regards to resistant microorganisms. This is in contrast to mandated tuberculosis screening, which overall has a significantly decreased impact on the healthcare system.

**• Showering or bathing with skin antiseptics:**

A Cochrane systematic review investigated the common practice of preoperative bathing/showering with skin antiseptics as a measure to reduce SSIs. They looked at 7 randomized controlled trials with a total of more than 10,000 patients that tested chlorhexidine solution against normal soap or no preoperative washing. They found no evidence suggesting a clear benefit [51]. Accordingly, it may be wiser to spend effort on more effective interventions.

**• Antiseptic skin cloths:**

Another proposed preoperative intervention is the local application of antiseptic solution at the planned surgical site. In a prospective RCT, Murray et al. [52] investigated the efficacy of the home application of a 2% chlorhexidine gluconate (CHG) cloth before shoulder surgery in decreasing the skin surface levels of bacteria. The overall positive culture rate in the treatment group vs control group was 66% vs 94% (p = 0.0008). The positive culture rate for coagulase-negative Staphyloccocus was 30% vs 70% (p = 0.0001) [52]. However, there were no infections in either group, so they were unable to directly correlate the reduction in culture rates with infection rates. Nevertheless, the culture rates do suggest a potential benefit, especially at the low cost of $3/package of 2 chorhexidine gluconate cloths [52].

In terms of definitive SSI rates, Eiselt [53] demonstrated its reduction in orthopedic patients undergoing joint replacements. Patients used a 2% CHG no-rinse cloth the night before the surgery and in the preoperative area immediately before the surgery. The control group was historical, prior to the intervention, and included 727 patients, and the treatment group, prospective, included 736 patients. A significant reduction in SSI rate was demonstrated after the implementation of CHG cloths (3.19% vs. 1.59%) [53].

Likewise, Graling et al. [54] conducted a prospective cohort study that included 284 patients as a historical control vs 335 patients who received CHG cloth intervention. The overall infection rate was decreased from 6.3% (18/284) to 2.1% (7/335) (p = 0.01). Their economic analysis differed slightly from Murray's. They estimated a total financial burden of $7 per patient, allotting $2/patient for nursing time for patient education and assistance. This is still considerable cheaper than the costs associated with patient morbidity and increased length of stay due to a SSI (estimated at ~ $25,000 per SSI) [55].

**• Hair removal:**

A Cochrane systematic review investigated the routine practice of preoperative hair removal. They looked to determine if routine removal compared to no removal and the timing or methods of removal influenced the rate of SSIs. 14 trials were included. No statistically significant evidence was found that indicated hair removal influences SSI rate; however, evidence did suggest that if hair removal was necessary to facilitate surgery or application of adhesive dressings, clipping compared with shaving reduces the rate of SSIs [56]. Three trials (1343 participants) compared the two, and demonstrated significantly more SSIs associated with shaving than clipping (Relative Risk 2.09, 95% CI 1.15 to 3.80) [56] (Table 1).

**Table 1:** **Summary of preoperative interventions**

Preoperative interventions	Summary	References
Screening for MSSA and MRSA	• Screening and prophylactic decolonization may prevent nosocomial S. aureus infections in carriers	[47]-[49]
Decolonizing hospital personnel	• Preliminary study finds reduction of SSI rate with decolonization of hospital personnel carriers	[50]
Showering or bathing with antiseptics	• Cochrane review of 7 RCTs finds no evidence of clear benefit	[51]
Antiseptic skin cloths	• Preoperative application of CHG cloths on planned surgical sites decreases skin surface levels of bacteria and may reduce SSI rate	[52]-[54]
Hair removal	• Cochrane review finds no evidence that hair removal has any bearing on SSI rates	[56]
	• However, clipping, compared to shaving, is associated with decreased incidence of SSIs	

### Intraoperative protocols

**• Antibiotics:**

Prophylactic administration of antibiotics has been proven effective in reducing the rate of postoperative infections for orthopedic, neurological, and spinal surgeries. In a meta-analysis of randomized controlled trials (RCTs) of spine fusion surgery, Baker et al. reported a significant reduction in SSIs, up to 63%, odds ratio 0.37 (95% CI 0.17-0.78) [57]. Similarly, other studies have substantiated the efficacy of perioperative antibiotics in general orthopedics, total joint replacement, and spinal surgery [58],[59].

Several guidelines exist for the prophylactic administration of antibiotics. Generally, they advocate a broad-spectrum antibiotic with excellent coverage of S. aureus such as a first or second-generation cephalosporin (e.g. cefazolin or cefuroxime). For those with beta-lactam allergies, clindamycin or vancomycin should be administered instead. Moreover, those who are at high risk of colonization with MRSA or have had a previous MRSA infection should be considered for prophylaxis with vancomycin.

As for timing and duration, high serum and tissue levels of antibiotic should be sufficiently obtained prior to the first incision. Therapy should be initiated within one hour prior to incision, and stopped within 24 hours of closure. Duration greater than 24 hours may lead to superinfection with drug-resistant organisms [60]. For surgical procedures that are prolonged > 4 hours or incur > 1500 mL of estimated blood loss, a re-dosing is recommended [61]. Lastly, the compliance of timing, duration, and selection of antibiotics is improved from 65% to 99% if the protocol is incorporated into the "time-out" [62].

**• Skin preparation:**

Prior to incision, the surgical site is often prepared by sterilizing the skin. Most commonly, a commercial skin antiseptic solution is applied such as Chloraprep (2% chlorhexidine gluconate and 70% isopropyl alcohol), DuraPrep (0.7% iodine and 74% isopropyl alcohol), or Betadine (0.75% iodine scrub, 1.0% iodine paint). Several studies have been conducted to compare the efficacy of these common preparation solutions. Ostrander et al. reported that ChloraPrep was superior to DuraPrep and Technicare in terms of eradicating bacteria from the skin, with decreased rates of positive cultures (30% vs. 65% vs. 95%, respectively, p < 0.0001) [63]. On the other hand, Savage et al. found no statistically significant difference in the rate of positive cultures between ChloraPrep and DuraPrep in a prospective study of 100 consecutive patients undergoing lumbar spinal surgery (0% vs 6%, p = 0.25) [64].

As for infection rates, a prospective cohort study that enrolled 3,209 patients found that DuraPrep was associated with the lowest rate compared with Betadine and ChloraPrep (3.9% vs. 6.4% vs. 7.1%, p = 0.002) [65]. Conversely, a multicenter prospective RCT reported ChloraPrep was associated with a lower rate of SSI than Betadine (9.5% vs. 16.1%, p = 0.004, risk ratio 0.59, 95% CI 0.41-0.85) [66].

As it stands, there is no clear evidence that one preparation solution is the better choice in effectively lowering rate of SSI.

**• Wound irrigation:**

Wound irrigation is one of the oldest surgical mantras, and many a medical student has heard their attendings singsong "dilution is the solution to pollution". Irrigation helps to remove loose, necrotic tissue, particulate debris, and microorganisms from within the surgical site. It is considered the most important intraoperative step in reducing the risk of infection. Traditionally, sterile normal saline has been used despite concrete, supporting evidence [67]. In fact, one prospective, randomized, double-blinded controlled study found that there was no statistically significant difference in rates of SSI between irrigation with saline vs tap water [68].

Irrigation with other solutions has also been suggested. One prospective RCT looked at the efficacy of dilute betadine irrigation (3.5% povidone-iodine solution) in the prevention of postoperative infection in spinal surgery. The study demonstrated a remarkably lower infection rate with dilute betadine solution (0%, n = 208) versus normal saline irrigation (3.5%, n = 206) (p = 0.007) [69]. No adverse side effects or events were reported.

**• Thermoregulation:**

Intraoperative hypothermia has been shown to increase the risk of SSIs, likely as a consequence of the reduction in peripheral circulation [70]. This reduction ultimately limits oxygen concentrations in the tissues, especially in the wound, where the body needs it most to fight off infections. Moreover, hypothermia directly impairs immune function. Warming patients have been demonstrated to reduce infection rates in colorectal surgery [70]. Melling et al. [71] looked at warming patients before clean surgeries (locally or systemically) and found the infection rate was 14% in non-warmed patients (19/139) versus 5% (13/277) in warmed patients. Among warmed patients, the study did not demonstrate a statistically significant difference in infection rates between local and systemic warming.

These studies suggest that maintenance of perioperative normothermia may be a worthwhile endeavor, especially since it is easily implemented.

**• Antiseptic-coated sutures:**

Sutures coated with the antiseptic triclosan have been developed to reduce SSIs. Edmiston et al. [72] evaluated its effectiveness in inhibiting bacterial growth and adherence in an *in vitro* model. There was > 75% reduction in gram-positive and gram-negative bacterial adherence to the antimicrobial suture. Clinically, in a prospective, double-blinded RCT in pediatric neurosurgical patients, Rozzelle et al. [73] demonstrated a statistically significant reduction in infection rates in those treated with antimicrobial suture (4.3% vs 21%, p = 0.038) undergoing cerebrospinal fluid shunt procedures. Unfortunately, the cost-effectiveness of these sutures is yet to be determined. The antiseptic-coated sutures cost 7% to 10% more than standard uncoated ones. Nevertheless, they may be justified in high-risk patients, as was the case with Rozzelle's patients, in which they underwent a procedure usually associated with 5-15% risk of infection [73].

**• Operating room traffic:**

Traffic in and out of the OR increases bacterial counts, and has been demonstrated to increase infection rates. The opening and closing of doors likely disrupts the airflow, allowing microbes to settle in the air directly above the surgical field. Panahi et al. [74] looked at the incidence of door opening during primary and revision total joint arthroplasty procedures. The study found that door openings averaged 0.65 and 0.84 per minute for primary and revision procedures, respectively. The average total door openings per procedure were 60 in primary cases and 135 in revisions [74].

The intervention here is clearly to limit traffic flow. This requires education and detailed communication among surgeons and OR personnel to aid preparation of the OR with essential instruments and components for scheduled procedures (Table 2).

**Table 2:** **Summary of intraoperative interventions**

Intraoperative interventions	Summary	References
Prophylactic antibiotics	• Prophylactic antibiotics are effective in reducing SSI rates	[57]-[62]
	• Discontinue within 24 hours of closure to prevent superinfection	
	• Redosing may be beneficial for procedures > 4 hours or if EBL > 1.5 L	
	• Timing, duration, selection of antibiotics should be incorporated into the "time-out" to improve compliance	
Skin preparation	• No clear evidence that one preparation solution is superior	[63]-[66]
Wound irrigation	• Important to remove loose, necrotic tissue, debris, and microorganisms from surgical site	[67]-[69]
	• No difference in infection rates between saline and tap water	
	• Dilute betadine solution may be of benefit	
Thermoregulation	• Maintaining perioperative normothermia may reduce SSI rates	[70],[71]
Antiseptic-coated sutures	• Effective in reducing SSI rates in neurosurgical patients, but cost-effectiveness has yet to be determined	[72],[73]
Operating room traffic	• Limit OR traffic flow	[74]

### Postoperative management

**• Drains and blood transfusions:**

A Cochrane review evaluated the occurrence of infections in relation to closed suction drainage after orthopedic surgery. 36 studies were included, involving 5464 participants with 5697 surgical wounds identified [75]. The meta-analysis demonstrated no statistically significant difference in infection rates between those with drains and those without. They concluded that there is no clear evidence that supports the routine use of closed suction drains in orthopedic surgery.

The aforementioned study did however find that drain use was often associated with the need for blood transfusions. Of course, blood transfusions carry its own risk of infections with blood-borne bacteria, viruses, or parasites – albeit a very minimal one. The more pressing risk associated with blood transfusions is the increased length of hospital stay and SSI [76]. It is thought that transfusion evokes an immunomodulation, which affects wounds and increases risk of infection [77]. Bower et al. [78] found that patients who received transfusions were nearly twice as likely to have an infection than those that did not. More studies are needed to pin down whether it's a causal relationship or merely a confounding finding such that people who are at risk of needing a blood transfusion are equally at risk for SSIs.

Regardless, blood transfusions do lead to an increased length of stay as demonstrated by Weber et al. [76]. This in it of itself is a risk factor for infection, and every effort should be made to judiciously discern the need for a transfusion. Preoperative assessment of hemoglobin and hematocrit levels and monitoring for symptomatic anemia rather than lab results alone would be beneficial.

**• Wound management:**

Postoperatively, the goal is to keep the surgical site clean and dry. The CDC recommends surgical dressings for 24–48 hours [79]. However, any soiled or blood-soaked dressings should be replaced immediately. Otherwise, any microorganisms nearby can become a source of infection. Dressings should also be chosen carefully to ensure they stay intact.

Antimicrobial dressings are available and they may serve to reduce risk of infection. Silver-based dressings, in particular, have been shown to be effective in reducing the rate of mediastinitis following cardiac surgery [80].

Another common wound dressing is the use of negative pressure wound therapy. There is not much literature on whether it helps decrease risks of infection. But by drawing out fluid from the wound, completely sealing the wound, and cutting down the frequency of dressing changes, theoretically it should help reduce the incidence of SSIs.

**• Urinary tract infections:**

Although not strictly a SSI, urinary tract infections are important to mention in the context of postoperative management, since it is a common complication. The details, however, are beyond the scope of this review. Some important principles to keep in mind are the following: avoid catherization when possible and remove as soon as possible; always practice aseptic technique during insertion and maintenance; consider the use of suprapubic and condom catheters in lieu of urethral catheters, since they have a lower rate of infection [81]. Finally, in high-risk patients, silver alloy catheters may have some antimicrobial benefit [82] (Table 3).

**Table 3:** **Summary of postoperative interventions**

Postoperative interventions	Summary	References
Drains and blood transfusion	• Cochrane review finds no difference in infection rates between those with drains and those without	[75]-[78]
	• Blood transfusions increase risk of infection and length of hospital stay	
Wound management	• Keep surgical dressings clean and dry Antimicrobial dressings may help reduce infections	[79],[80]
Urinary tract infection	• Avoid catherization when possible, and always remove as soon as possible	[81],[82]
	• Suprapubic and condom catheters have lower rates of infection	
	• In high-risk patients, silver alloy catheters may be of benefit	

### Non-operative preventive measures

Briefly, we would like to mention some other preventive measures. These are not specifically related to SSIs but are more general measures. The multifactorial etiology of health-care associated infections precludes successful prevention in a vacuum of its subtypes (i.e. SSIs, UTIs, BSIs, PNEUs), so it is important to be cognizant of these other measures. For more information, Curtis provides a detailed review on a number of these interventions [83].

**• Cleaning:**

Proper cleaning is an important aspect of preventing health-care associated infections. Contaminated environmental surfaces often lead to cross-transmissions, and increase rates of infection. Hospitals should train cleaning personnel adequately, monitor performance regularly, and provide feedback. Moreover, they should make hospital cleaning personnel aware of their vital role in the ultimate health of the patients. A prospective study in Illinois compared rates of VRE infection before and after a cleaning educational program. Rates of VRE infection fell by 64% [95% CI: 0.19-0.68] [84].

There are few studies looking at which chemicals are best to clean surfaces. However, some studies demonstrate the effectiveness of a bleach solution. One study compared the use of 1:10 hypochlorite (bleach) solution with the use of a quaternary ammonium solution and found that the former was associated with a significantly lower rate of *C. difficile* infection than the latter. Another study found that unbuffered 1:10 hypochlorite solution reduced the frequency of positive *C. difficile* cultures in patient rooms from 31% to 16%. Finally, a study of 17 rooms that housed VRE-positive patients found that prior to cleaning, 16/17 (94%) of the rooms contained viable VRE. After thorough cleaning with a 10% bleach solution, the amount of viable VRE decreased to 0 (0%) (p < 0.001) [85].

Hydrogen peroxide vapor has also been shown to be an effective decontamination method. A British study compared manual cleaning with hydrogen peroxide vapor. Prior to manual cleaning, in 10 surgical ward rooms, 89% of 124 swab samples were positive for MRSA and 66% remained positive after manual cleaning. In comparison, 6 other surgical ward rooms were swabbed and 72% of 85 swabs were positive prior to cleaning. After hydrogen peroxide treatment, only 1% of 85 remained positive [86]. Another study investigated the feasibility of routine hydrogen peroxide decontamination in a busy hospital. One drawback has always been the mean time for decontamination with hydrogen peroxide (~90-120 minutes). The study concluded that the additional time is offset by the drastic improvement in surface hygiene, and reduction in nosocomial pathogens. They assert its feasibility in a busy hospital with a mean occupancy rate of 94% [87].

**• Waterborne transmission:**

Studies have found that the replacement of tap water with sterile water for drinking, bathing, and procedures can significantly reduce infection rates [19]. Other measures include decontaminating the hospital water supply. This can be done by heating water to more than 50°C or with UV light treatment, both of which has been shown to reduce levels of *Legionella*[88],[89]. Another effective method is copper-silver-based ionization systems, has been shown to reduce molds and gram-negative bacteria such as *P. aeuroginosa* and *Actinetobacter baumannii* in addition to *Legionella*[20],[90],[91]. One hospital found legionella infection rates drop from 2.45 cases to 0.18 cases per 1000 discharges subsequent installation of a copper-silver ionization system [91].

**• Air filtration/treatment:**

High-efficiency particulate air filters can help reduce aerosolized pathogens and decrease infection rates. Studies have reported its use decreases airborne aspergillus concentrations and aspergillus infections [92],[93]. Moreover, the use of portable filters has been demonstrated to significantly reduce airborne levels of MRSA and *P. aeruginosa*[94]*.*

Adequate outdoor air ventilation is also important to help circulate and dilute air that may be ridden with airborne pathogens. Indeed, poor ventilation is often associated with higher rates of acute respiratory disease [95].

**• Anti-microbial copper alloy:**

As mentioned above, touch surfaces are often a source of contamination and cross-transmission. Surfaces can often harbor pathogens for days and even months, becoming a reservoir of infection. Incorporating anti-microbial copper alloy into these surfaces has been suggested as a possible solution. Copper's basic chemical properties and tendency to produce hydroxyl radicals and cations gives it a unique broad-spectrum biocidal ability. In vitro studies have demonstrated its effectiveness in rapidly reducing bacterial concentration by 7 logs within just 2 hours [96]-[98].

A multi-center study looked at its clinical application in the ICUs of 3 hospitals. Patients were randomly assigned to rooms with and without copper alloy surfaces, and the rates of HAIs and/or colonization with MRSA or VRE were compared. Six objects were fabricated from copper alloy, four of which were identical among the three hospitals: bed rails, overbed tables, IV poles, and arms of visitor's chair. Results indicated that the rate of HAI and/or MRSA or VRE colonization in copper rooms were significantly lower than non-copper rooms (0.071 vs. 0.123, p = 0.020). The rate for just HAI was decreased 58% from 0.081 to 0.034 (p = 0.013) [98].

Another study found that the combined burden of MRSA and VRE were 96.8% lower on copper surfaces than current plastic, wood, metal, and painted surfaces. Copper surfaces were also found to continuously limit microbial bioburden, achieving same levels as terminal cleaning [99]. Copper-alloy surfaces are proving to be a promising intervention (Table 4).

**Table 4:** **Summary of non-operative preventive measures**

Non-operative preventive measures	Summary	References
Cleaning	• Hospital cleaning personnel should be made aware of their vital role in decreasing HAIs; they should be trained appropriately, adequately, and monitored	[84]-[87]
	• Bleach solutions and hydrogen peroxide vapor have been shown to be effective decontaminating agents	
Waterborne transmission	• Replacement of tap water with sterile water may reduce infection rates	[88]-[91]
	• Decontaminating hospital water supply may be an alternative	
Air filtration	• High-efficiency particulate air filters can help reduce airborne levels of aspergillus, MRSA, and P. aeruginosa	[92]-[95]
Anti-microbial copper alloy	• Incorporating copper-alloy in hospital surfaces may limit contamination and cross-transmission	[96]-[99]

### Developments

At Brigham and Women's Hospital, in the Laboratory for Tissue Repair and Gene Transfer, we have developed a wound enclosure device, which we have previously shown to be a promising wound healing modality without any adverse side effects [100]-[103]. It consists of a polyurethane chamber that envelops the wounds, creating a controlled incubator-like microenvironment. This platform allows us to maintain optimal conditions for wound healing while concomitantly providing both topical antibiotics and analgesics to the wound [100]-[103]. In addition to wound healing improvement, we have realized its potential as a novel intervention for health-care associated surgical site infections. These chambers eliminate the need for daily dressing changes, since fluid can easily be aspirated and sampled from the chamber itself, and the clear chamber allows easy visual inspection of the wounds, facilitating hospital rounds. This would significantly decrease the risk of contamination from hospital personnel.

Using a porcine model, we have been able to dramatically decrease bacteria concentrations in both wound fluid and tissue by adding topical antibiotics (up to 1,000 minimal inhibitory concentration) within our wound chambers, even after inoculation of wounds with 10^8^ colony forming units/mL (unpublished data). Moreover, our preliminary studies indicate its ability to rapidly decrease bacterial counts of endogenous flora. This has great implications, since, as mentioned, endogenous flora is a common source of contamination that leads to SSI. Similar to the antiseptic skin cloths discussed above, which have been shown to reduce infection rates, these chambers may be a safe, more efficacious alternative. Theoretically, these chambers could be placed preoperatively on the surgical site to rapidly decolonize endogenous flora, and also be placed as a wound dressing post-operatively to further prevent SSIs. Further studies are planned, but at this point, it looks to be a promising novel intervention that could easily be implemented.

### Numbers and costs

Using data from the National Nosocomial Infections Surveillance (NNIS) system, National Hospital Discharge Survey (NHDS), and the American Hospital Association (AHA) Survey, Klevens et al. estimated 1.7 million HAIs occurred in U.S. hospitals in 2002. Among these, approximately 155,000 deaths occurred, 99,000 of which were considered caused by or significantly associated with the HAI [3]. This puts HAIs in the top ten leading causes of death, compared to CDC's 2010 data for Alzheimer's disease (83,494), diabetes (69,071), and kidney diseases (50,476) [104]. In another study, Scott et al. estimated that the annual direct cost of HAIs to U.S. hospitals ranges from $28.4 to $45 billion [4]. With a high estimate that 70% of HAIs are preventable by current available strategies, the economic benefits of prevention ranges from $25.0 to $31.5 billion. With a low estimate that 20% are preventable, the savings range from $5.7 to $6.8 billion, which is still comparable to the healthcare costs of stroke ($6.7 billion), diabetes mellitus with complications ($4.5 billion), and chronic obstructive lung disease (4.2 billion) [105].

Surgical site infections alone account for roughly 290,000 of the total HAIs, and are estimated to cause 8,000 deaths, a case fatality rate of 2.8%. They are the second most frequently reported HAI [3]. As for the attributable costs of SSIs, Anderson et al. gives a low estimate of $10,443 per infection and Stone et al. gives it a high estimate of $25,546 per infection [55],[106]. From a patient perspective, wound infections are the second most commonly experienced adverse event (14%), second only to medication errors (19%) [107]. Commonly, SSIs increase the hospital length of stay (LOS) on average between 7.3 days and 14.3 days [108]. Moreover, they increase the risk of re-admittance within 30 days by 5 times, double the mortality rate, and decrease quality of life [109],[110]. In a systematic review, Umscheid et al. estimated that as much as 55% of SSIs may be preventable. This would equate to approximately 75,526 to 156,862 preventable infections per year and subsequently, 2,133 to 4,431 preventable deaths [111].

### Final thoughts

Surgical site infections remain a tremendous issue in the 21st century. There are several successful preventive measures described in the literature; however, an institution-wide adoption of several of these interventions is often few and far between. Complacency in this matter is almost tantamount to negligence. In the spirit of *primum non nocera*, we should ever strive to aggressively lower infection rates as much as we can. Additionally, with the rapidly changing climate of our healthcare industry, where the way we practice and the quality of our care is increasingly scrutinized, infection prevention should be an utmost priority.

Although, it is doubtful one single preventive measure will ever be the be-all and end-all for our problems, there is hope that bundles of multiple measures can dramatically reduce the rate of SSIs – a testament to Aristotle's "the whole is greater than the sum of its parts" [112]-[114]. Alexander et al. suggests that adherence to current guidelines and available interventions can reduce infection rates to less than 0.5% in clean wounds, less than 1% in clean contaminated wounds, and less than 2% in highly contaminated wounds [115]. With a similar mindset, the Mayo Clinic in Florida instituted multiple interventions as an SSI Bundle. Thompson et al. [116] details their institution's experience and results in their paper aptly titled, "Chasing Zero". During the study period between May 2008 and June 2010, they achieved a 57% decrease in SSI rate with an estimated savings of nearly $1 million (Class I SSI, clean wounds, rate from 1.78% to 0.51% and Class II SSI, clean-contaminated, rate from 2.82% to 1.44%) [116].

## Conclusion

Health-care associated infections continue to cause significant patient morbidity and mortality and account for a great deal of healthcare costs. Nevertheless, we are optimistic that together we are ever moving towards improving patient care. Though many successful preventive measures exist, more can always be done in terms of research and practicing evidence-based care. We believe that pushing for an organized institution-wide adoption of multiple interventions may be the key to reducing infection rates. No doubt each institution differs in capacity and resources, so a discussion is necessary at each institution on what is feasible. We hope this review and the work of many others before us can help inspire additional dialogue regarding this matter.

## Abbreviations

HAI: Healthcare-associated infection
SSI: Surgical site infection
CDC: Centers for disease control
NHSN: National healthcare safety network
UTI: Urinary tract infections
BSI: Bloodstream infections
PNEU: Pneumonia
MRSA: Methicillin-resistant staphylococcus aureus
VRE: Vancomycin-resistant enterococci faecium
CHG: Chlorhexidine gluconate
RCT: Randomized controlled trial
